# Clinical presentation, magnetic resonance imaging findings, and outcome of 80 Dachshunds with cervical intervertebral disc extrusion

**DOI:** 10.3389/fvets.2024.1438300

**Published:** 2024-08-29

**Authors:** Francesca Violini, Federica Tirrito, Francesca Cozzi, Barbara Contiero, Simone Anesi, Eric Zini, Cristina Toni

**Affiliations:** ^1^Willows Veterinary Centre & Referral Service, Part of Linnaeus Veterinary Limited, Solihull, United Kingdom; ^2^AniCura Istituto Veterinario Novara, Novara, Italy; ^3^Studio Veterinario Associato Vet2Vet Ferri e Porporato, Torino, Italy; ^4^Clinica Neurologica Veterinaria – NVA, Milan, Italy; ^5^Dipartimento di Medicina Animale, Produzioni e Salute – Università degli Studi di Padova, Padua, Italy; ^6^Clinic for Small Animal Internal Medicine, Vetsuisse Faculty, University of Zurich, Zürich, Switzerland

**Keywords:** intervertebral disc extrusion, Dachshund, cervical disc extrusions, spinal surgery, MRI

## Abstract

**Introduction:**

Large clinical studies regarding cervical intervertebral disc extrusion (IVDE) in Dachshunds are lacking. This retrospective multicentric study therefore aims to describe the clinical features, magnetic resonance imaging (MRI) findings and outcomes of Dachshunds diagnosed with cervical IVDE.

**Methods:**

Medical records of Dachshunds with cervical IVDE were reviewed for signalment, onset of clinical signs, neurological examination, MRI features, treatment and outcome.

**Results:**

Eighty Dachshunds were included in the study, mostly ambulatory (55% grade 1 and 33% grade 2) and without nerve root signature (85% of cases) on presentation. Information on coat type was available for 56% of dogs; specifically, 41% were smooth-haired, 9% were long-haired and 6% were wire-haired Dachshunds. There were 29 (36%) neutered female, 27 (34%) male entire, 15 (19%) male neutered and 9 (11%) entire female dogs. The onset of clinical signs was most often >48 h (84%). The most common intervertebral disc space affected was C2-C3 (38%) and foraminal IVDEs were reported in 14% of dogs. A foraminal IVDE was diagnosed in only 25% of dogs presented with nerve root signatures. Most dogs (77.5%) were treated surgically. In this group, a higher body condition score on presentation and a higher mean spinal cord compression ratio calculated on MRI were directly and moderately associated with a longer hospitalization time (*r* = 0.490 *p* = 0.005 and *r* = 0.310 *p* = 0.012, respectively). The recovery time was longer in dogs with an onset of clinical signs <24 h or 24–48 h compared to those with an onset of clinical signs >48 h (3.1 ± 6.5 days versus 1.6 ± 6.2, *p* < 0.001) in both medically and surgically treated groups. Data about the outcome was available for 83% of dogs. Eighty percent of the entire population of dogs was considered to have completely returned to normal. There was no association between the therapeutic choice (surgical versus medical management) and the outcome of the dogs included in this study.

## 1 Introduction

Intervertebral disc disease is one of the most common neurological disorders in dogs. Chondrodystrophic breeds, such as Dachshunds, are over-represented and are most commonly affected by thoracolumbar intervertebral disc extrusions (1, 2). Cervical intervertebral disc herniation accounts for up to 25.4% of all cases of intervertebral disc herniation (3), and intervertebral disc extrusion (IVDE) appears more frequent than intervertebral disc protrusion in dogs (4). Chondrodystrophic breeds represent the majority of cases of cervical intervertebral disc herniation (5). Among these chondrodystrophic breeds, Dachshunds account for approximately 35–36% of cases of cervical intervertebral disc herniation (4, 5).

The predominant clinical sign of cervical IVDE is usually marked cervical hyperesthesia which may be acute or chronic in presentation (6). Often, no further neurological deficits are witnessed and this is possibly the result of the large vertebral canal to spinal cord ratio in the cervical vertebral column (3). Another possible finding associated to cervical hyperesthesia is the “nerve root signature,” defined as radiating pain associated with a nerve root entrapment resulting in lameness of variable severity (7). This has been described in association to extrusion of intervertebral disc into the caudal cervical intervertebral foramina in 15–50% of cases (3).

The clinical course, treatment and prognosis of IVDE has already been described in different dog breeds, both for thoracolumbar [French bulldogs and Dachshunds in Aikawa et al. (8); English Cocker Spaniels in Cardy et al. (9); Penkingeses in Chai et al. (10)] and for cervical IVDE [Yorkshire Terriers in Palus et al. (11)]. To the authors’ knowledge, there are no previous studies in veterinary literature specifically addressing cervical IVDE in Dachshunds. This retrospective multicentric study proposes the following objectives: (a) to describe the clinical features and imaging findings of Dachshunds diagnosed with cervical IVDE; (b) to determine the prevalence of cervical IVDE in the population of neurologically affected Dachshund based on sex, age, and phenotype; (c) to compare the outcome of medically versus surgically treated cervical IVDEs in Dachshunds.

## 2 Materials and methods

Medical records of two referral centers, Willows Veterinary Centre and Referral Service (Birmingham, United Kingdom), and Clinica Neurologica Veterinaria NVA (Milan, Italy) were retrospectively reviewed for Dachshunds diagnosed with cervical IVDE between August 2010 and August 2023. Data collected included signalment, onset of clinical signs and neurological examination findings.

Signalment information included age, sex, neutering status, size, coat type and color, weight and body condition score [on a scale from 1 to 9, as described by Laflamme (12)]. Size was recorded (based on the information provided by the owner) as kaninchen, miniature and standard (as recognized by the Federation Cynologique Internationale – FCI). The coat type was divided into smooth, long-haired and wire-haired. The color was divided into red, black, chocolate, dapple, cream, wild boar and chocolate wild boar.

Time from the onset of clinical signs to the original presentation at the referral practice was recorded and classified as <24 h, 24 to 48 h or > 48 h prior to the initial presentation as previously described (13, 14). Information regarding previous spinal problems and whether these were affecting the cervical vertebral column, or a different area were retrieved.

The presence or absence of serious comorbidities was also recorded. We defined “serious comorbidities” as any life-threatening condition or chronic illness requiring the administration of long-term medications.

The neurological examination findings at the time of presentation were categorized using a grading system adapted from previous literature (15, 16) where 0 = normal neurological examination, 1 = normal gait with cervical hyperesthesia, 2 = ambulatory tetraparesis, 3 = non ambulatory tetraparesis and 4 = tetraplegia. Grade 5 (i.e., tetraplegia with absent nociception) was purposely removed from the grading system as these patients suffer from a high mortality rate due to hypoventilation and brady-arrhythmias (17).

Neurolocalization (based on the neurological examination findings) as well as presence and side of nerve root signature were also documented. The neurological examination was always performed by an ECVN (European College of Veterinary Neurology) diplomate, by an ECVN-residency trained veterinarian or by an ECVN resident working under the supervision of an ECVN-diplomate.

### 2.1 Diagnostic imaging findings

The diagnosis of cervical IVDE was obtained via magnetic resonance imaging (MRI) scan of the cervical vertebral column which was available to be retrospectively reviewed. A diagnosis of compressive cervical IVDE was made based on previously reported criteria (18): (a) presence of extradural material causing spinal cord compression, (b) extradural material centered over or near an intervertebral disc space and (c) on T2-weighted (T2W) sequences, displacement or loss of the hyperintense signal of the cerebrospinal fluid within the subarachnoid space and of the epidural fat as a result of compression and/or displacement of the spinal cord by the extradural material. A diagnosis of foraminal IVDE was made when there was presence of extruded disc material within the foraminal space and there was no associated spinal cord compression (19). If a compressive cervical IVDE was accompanied by the presence of extradural material within the intervertebral foramen, this was described as a compressive IVDE with a foraminal component.

**Figure 1:**
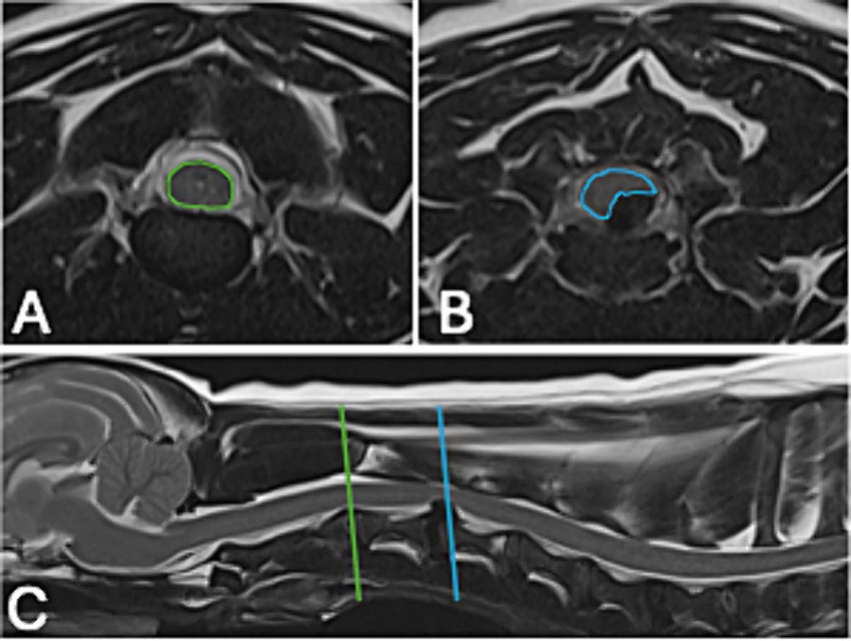
Example of the measurements used to calculate the spinal cord compression (SCC) ratio in one of the patients in our study. **(A)** T2W transverse at the level of intervertebral disc space immediately cranial to the area of compression, the area used for the spinal cord cross sectional area (CSA) has been highlighted in green. **(B)** T2W transverse at the point of maximal compression, the area used for the CSA has been highlighted in blue. **(C)** T2W sagittal of the cranial cervical vertebral column outlining the two areas used for **(A,B)**.

The MRI study was performed using a 1.5 tesla magnet (Magentom Sola, Siemens healthcare limited, Erlangen, Germany; Signa HDe, GE healthcare, United States), a 0.4 tesla magnet (Hitachi Aperto Lucent, Hitachi Medical Systems, Milan, Italy) and a 0.3 tesla magnet (Hitachi Airis II, Hitachi Medical Systems, Milan, Italy). The site of the IVDE was recorded, as well as the presence or absence of extruded intervertebral disc material in the intervertebral foramen, the lateralization of the extruded material, and the presence or absence of spinal cord compression. The signal intensity of the extruded intervertebral disc material on T2W sequences was described as homogenous if it had uniformly hypointense signal intensity compared to spinal cord grey matter, or heterogenous compared to spinal cord grey matter if the extruded disc material had mixed hyperintense or hypointense signal intensity (20). The presence or absence of intramedullary hyperintensity on T2W sequences was recorded. The degeneration of the herniated intervertebral disc was graded using the Pfirrmann grading system on T2W sagittal sequences, as previously described (18, 21). The details of the Pfirrmann grading system are summarized in Table 1. The degree of spinal cord compression caused by the extruded disc material was assessed by calculating the spinal cord compression (SCC) ratio as previously described (16). The spinal cord cross sectional area (CSA) was measured on T2W transverse images by tracing the outline of the spinal cord with an image analysis software tool at the point of maximal compression (Figure 1). The same procedure was repeated in an unaffected area, conventionally, this was done at the intervertebral disc space immediately cranial to the area of compression. The following formula was then used to calculate the SCC ratio:


$$\frac{\begin{array}{l} \mathrm{CSA}\;\mathrm{of unaffected spinal cord}-\\ \mathrm{CSA}\;\mathrm{of spinal cord under maximal compression} \end{array}}{\mathrm{CSA}\;\mathrm{of unaffected spinal cord}\;}\;\times \;100$$

**Table 1:** Pfirrmann grading system for intervertebral disc degeneration.

Grade	Appearance of the NP	Distinction between NP and AF
1	Homogenous, hyperintense signal intensity	Clear
2	Nonhomogeneous with or without horizontal bands, hyperintense signal intensity	Clear
3	Nonhomogeneous, intermediate signal intensity	Unclear
4	Nonhomogeneous, intermediate to hypointense signal intensity	Lost
5	Nonhomogeneous, hypointense signal intensity	Lost

NP, nucleus pulposus; AF, annulus fibrosus.

For each patient, the SCC ratio was calculated three times by two ECVN diplomates in Veterinary Neurology and Neurosurgery (F.T., C.T.) and then the average value was calculated. The average value was used as the SCC ratio for each patient.

### 2.2 Treatment options and outcome

Information regarding medical and surgical treatment was collected from the patients’ medical records. Medical (conservative) management involved analgesia and at least four weeks of confinement to a restricted area (such as a crate or small room without furniture) except for time dedicated to outdoor toileting or physiotherapy; during this time off-leash exercise, jumping on or off furniture and access to stairs was forbidden (22). In surgical cases, the procedure type (ventral slot versus dorsal laminectomy) and the qualification of the veterinary surgeon performing the procedure (ECVN diplomate, ECVN resident or other) were analyzed. If physiotherapy was performed by a qualified veterinary physiotherapist as part of medical treatment or post-operative treatment, it was recorded. The total days of hospitalization and the outcome at 3–7 days, 21–30 days and six months were evaluated for all patients. Outcome was evaluated using a scale from 0 to 4 as described above. This evaluation was performed by the same operators (ECVN diplomate, ECVN-residency trained veterinarian or ECVN resident working under the supervision of an ECVN-diplomate). If information on a patient’s outcome was lost at any of these three points, the patient was still included in the study and recorded as “lost to follow-up.” A “successful outcome” was defined as a patient able to walk without assistance and with conscious urination. A patient was considered able to walk without assistance when able to rise and perform ten, unassisted, consecutive, weight-bearing steps (11, 23). The days from the initial presentation to successful outcome were recorded for each patient when available. Additionally, for both medical and surgical cases it was recorded if the patient had recovered normal function (i.e., normal neurological examination and absence of cervical hyperesthesia) within 30 days.

The decision between medical versus surgical treatment was made at the time of diagnosis after discussing both treatment options with the owners. If a patient undergoing medical management failed to show any clinical improvement, worsened or if the patient suffered from a recurrence of cervical IVDE-related clinical signs, this was recorded. Timing of signs recurrence or deterioration (within 30 days from their original presentation or after 30 days from their original presentation) was also recorded. If the patient had died as a result of related or unrelated causes, it was recorded.

### 2.3 Statistical analysis

Descriptive statistics were used to illustrate the clinical features and imaging findings of Dachshunds diagnosed with cervical IVDE. Analysis of data was carried out by statistical software SAS 9.4 (SAS Institute; Cary, NC). The prevalence of cervical IVDE in the population of neurologically affected Dachshunds was calculated based on sex, age, and phenotype, and comparison between the outcome of medically versus surgically treated Dachshunds was performed. Categorical data were reported as counts and corresponding percentages were compared using two proportions z-test or k-proportions test. Associations between two categorical variables were assessed using chi-squared tests (P Yeats for 2×2 contingency tables); if there were fewer than five dogs in a category, Fisher’s exact test was used. Continuous variables were assessed for normality using the Shapiro–Wilk test. Median, interquartile range (IQR) and range were reported for skewed data, whereas mean ± standard deviation (sd) was reported for normally distributed data. Differences in median values were compared using non-parametric Mann–Whitney *U*-tests, whereas means were compared using t-Student test. Association between continuous data was assessed using Spearman rank correlation coefficient (*r*). Absolute *r* values >0.6 were considered consistent with a strong correlation while a moderate correlation was assigned to values >0.4. Significance was set at a *p*-value <0.05.

## 3 Results

### 3.1 Signalment and clinical presentation

Eighty Dachshunds met the inclusion criteria. There were 38 females (nine entire, 29 neutered) and 42 males (27 entire, 15 neutered). Median weight at presentation was 7.2 kg (range: 3.9–23). Information on phenotype (size, coat type and color) is summarized in Table 2. Information on coat type was available for 45 cases. In the sample population there was a higher percentage of dogs with smooth coat type (41%) and of neutered female and entire male dogs (36 and 34%, respectively). Median age at diagnosis was seven years (range: 1–14). Information on BCS was available for 43/80 (53%) of dogs, with the majority being 5/9 (74%).

**Table 2:** Summary of phenotype distribution in our population.

	n° subjects	%
Size		
Miniature	34	43%
Standard	43	54%
Not available	3	4%
Coat type		
Long	7	9%
Smooth	33	41%
Wire-Haired	5	6%
Not available	35	44%
Color		
Black and Tan	20	16.8%
Red	7	5.8%
Wild Boar	3	2.5%
Chocolate	2	1.7%
Brindle	1	0.8%
Brown	1	0.8%
Brown and Tan	1	0.8%
Dapple	1	0.8%
Sex		
FE	9	11%
FN	29	36%
ME	27	34%
MN	15	19%

FE, female entire; FN, female neutered; ME, male entire; MN, male neutered.

The majority of dogs (56/80; 70%) had not suffered from previous spinal problems and most dogs (78/80; 98%) did not present with any serious co-morbidities.

The most common clinical score at presentation was grade 1 (44/80; 55%) and grade 2 (26/80; 33%), followed by grade 3 and 4 (7/80, 9% and 3/80, 4% respectively). When looking at the neurolocalization, 34/80 dogs (42%) were classified as “cervical hyperesthesia only,” 26/80 dogs (33%) as “C1-C5 spinal cord segments” and 20/80 dogs (25%) as “C6-T2 spinal cord segments.” The presence of nerve root signature was only reported in 12/80 (15%) dogs; 6/12 (50%) had a neurolocalization to the “C6-T2 spinal cord segments,” 4/12 (33%) to the C1-C5 spinal cord segments” and 2/12 (17%) had no neurological deficits that allowed neurolocalisation therefore were classified as “nerve root signature with cervical hyperesthesia only.”

The onset of clinical signs was mostly >48 h (67/80; 84%) with a mean of 18 ± 19 days. An onset of clinical signs of <24 h was reported in 10/80 (13%) of cases while only 3/80 (4%) of patients presented with an onset of clinical signs between 24 and 48 h prior to presentation. Thirty-seven out of the 67 (55%) dogs presenting with an onset of clinical signs >48 h were classified as a grade 1, 25/67 (37%) as a grade 2, 3/67 (5%) as grade 3 and 2/67 (3%) as grade 4. All cases presenting with a duration of clinical signs <24 h were classified as grade 1. Dogs with a duration of clinical signs between 24 and 48 h all presented as a grade 2.

### 3.2 MRI findings

The most common site of cervical IVDE was C2-C3 (31/80, 38%), followed by C3-C4 and C4-C5 (both reported in 14/80, 17%, dogs), C5-C6 (12/80, 15%), C6-C7 (7/80, 9%) and C7-T1 (3/80, 4%). There was a significant difference between these sites of IVDE with a higher prevalence for C2-C3 (*p* < 0.001). A foraminal IVDE was reported in 18/80 (14%) cases. Foraminal IVDE was found in the left side, the right side and bilateral; however, there was a significantly higher prevalence for the left side (56% vs. 33% vs. 11%, *p* = 0.018). There was no prevalence for a specific site for foraminal IVDE in the cervical vertebral column. In 10/18 (55%) cases the foraminal component was associated with a compressive cervical IVDE. Only 25% (3/12) of the dogs that presented with nerve root signature were diagnosed with a foraminal IVDE on MRI. Fifteen out of the 18 (83%) dogs diagnosed with a foraminal IVDE did not display a nerve root signature on presentation.

The signal intensity of the extruded intervertebral disc material on MRI was described as homogenously T2W hypointense compared to grey matter in 64/80 (80%) cases. The presence of T2W intramedullary hyperintensity compared to grey matter was reported in 59/80 (74%) cases. The median SCC was 27.91% (range 5.87–59.7%). Median Pfirrman grade was 4 (range 2–5).

### 3.3 Treatment choice and outcome

A total of 62 dogs (77.5%) underwent surgical management while the remaining 18 dogs (22.5%) underwent medical management. Surgical management involved a ventral slot in 60 dogs and a dorsal laminectomy in the remaining two. Medical management involved non-steroidal anti-inflammatory drugs (NSAIDs) in ten dogs, steroids in six dogs and paracetamol in two. Tramadol (2/18), gabapentin (11/18), opioids (2/18), pregabalin (2/18) or diazepam (1/18) were also administered in the medically treated cases.

There was a significantly higher prevalence for dogs with a compressive IVDE without a foraminal component that were treated surgically (68/80; 85%, *p* < 0.001). Medical management was used in all eight cases of purely foraminal IVDE. In 7/10 (70%) of the dogs where the foraminal component was associated with a compressive cervical IVDE, surgery (ventral slot) was performed. The remaining 3/10 cases were managed medically. Treatment choice was not influenced by the onset of clinical signs (*p* = 0.758). MRI findings such as signal intensity of the extruded intervertebral disc material (*p* = 0.547) or the presence of T2W intramedullary hyperintensity (*p* = 0.456) also did not seem to affect the treatment decision (i.e., medical versus surgical). Treatment choice was also not influenced by the degree of SCC (23 ± 9 vs. 30 ± 11, *p* = 0.072).

Median length of hospitalization was one day (range 0–9 days). Patients treated surgically spent on average a longer time in the hospital compared to medically treated dogs [median 2 days (range 0–9) versus 0 days (range: 0–4), *p* = 0.003]. In the surgical group, a higher BCS on presentation and a higher mean SCC calculated on MRI were directly and moderately associated with a longer hospitalization time (*r* = 0.490, *p* = 0.005 and *r* = 0.310, *p* = 0.012, respectively). Age at presentation was inversely and poorly correlated to the length of hospitalization for dogs treated surgically (*r* = −0.36, *p* = 0.004). For both medically and surgically treated dogs, a higher clinical score at presentation was directly moderately associated with a longer hospitalization (surgically treated group *r* = 0.360 and *p* = 0.004, medically treated group *r* = 0.470 and *p* = 0.047). In the comparison between surgically and medically treated dogs, the latter had significantly shorter hospitalization for neutered dogs [median 0 days (range: 0–4) versus 2 (range: 0–9), *p* = 0.004], for standard Dachshunds [median 0 days (range: 0–0), versus 1 (range: 0–5), *p* = 0.001], for patients without a foraminal disc extrusion [median 0 days (range: 0–1) versus 2 (range: 0–9), *p* = 0.002], for patients without T2W intramedullary hyperintensity [median 0 days (range: 0–4), versus 1 (range: 0–5) *p* = 0.007] and for patients with a homogeneously T2W hypointense extruded intervertebral disc material [median 0 days (range: 0–4) versus 2 (range: 0–9), *p* = 0.002].

**Figure 2:**
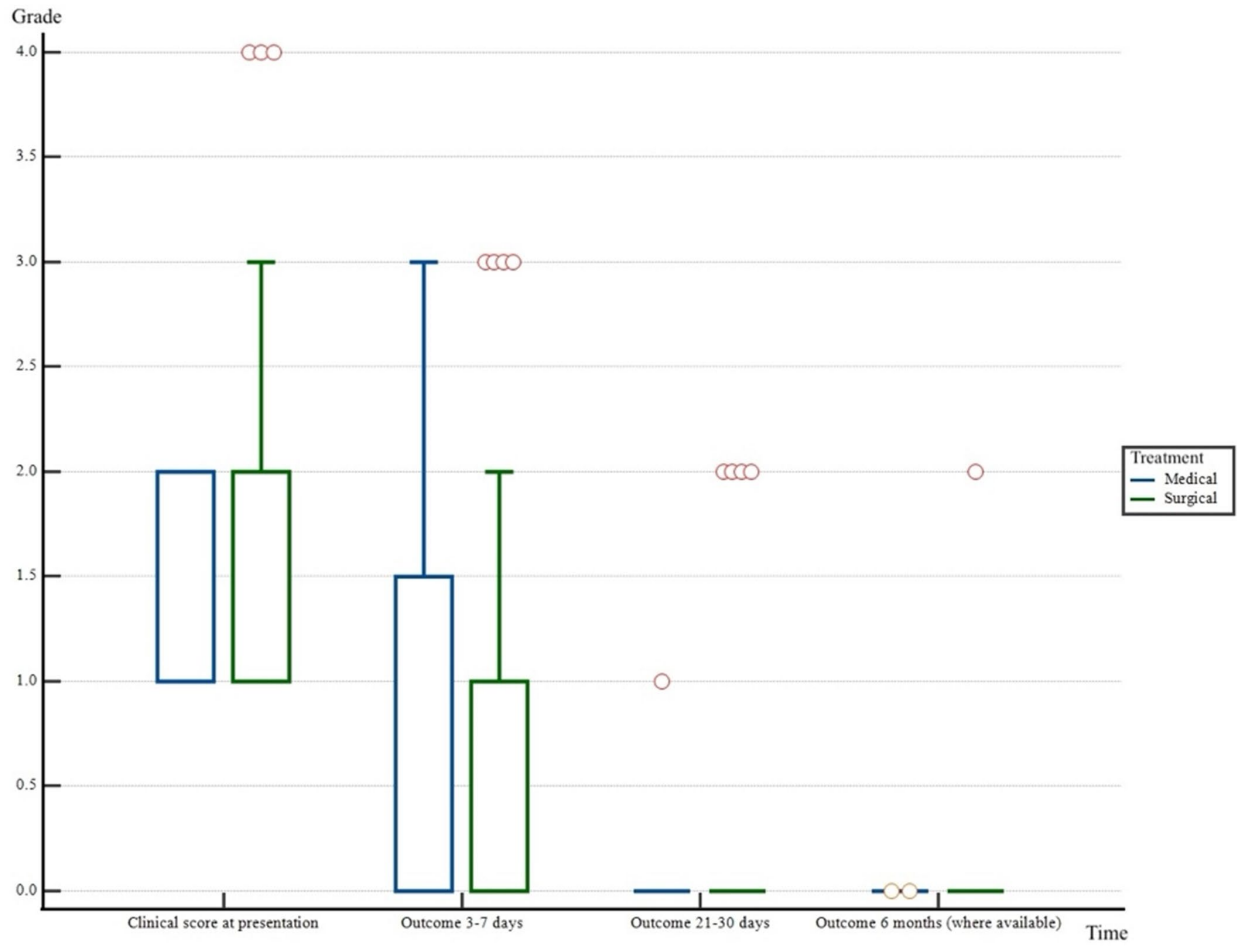
Boxplot representing neurological grade of the patients included in the study at the different check points (at presentation, at 3–7 days, at 21–30 days and at 6 months from diagnosis). x = different time points considered. y = neurological grade (from 0 to 4). ° = outliers.

Information on the outcome at 3–7 days from diagnosis and recovery time was available for 89% (71/80) dogs. Clinical score at 21–30 days from the initial diagnosis was obtained in 80% (64/80) dogs. Data regarding the six-month follow-up was available only for 15% (12/80) dogs. There was no significant difference between the therapeutic choice (surgical versus medical) for the initial neurological grade and the outcome at any of the time intervals mentioned above. Data has been summarized in Table 3 and Figure 2. The neurological grade in dogs at 21–30 days interval post-diagnosis was significantly higher for dogs with a purely foraminal IVDE compared to dogs with spinal cord compression on MRI [median 0 (range: 0–1) versus 0 (range: 0–0), *p* < 0.001]. In surgically treated patients, a longer (> 48 h) onset of clinical signs was moderately associated with a better outcome at 3–7 days (*r* = −0.430, *p* < 0.001). A higher neurological grade on presentation was strongly associated with a higher neurological grade at 3–7 days both for surgically and medically treated patients (*r* = 0.67 and *p* < 0.001, *r* = 0.63 and *p* = 0.028, respectively). There was a moderate association between a higher clinical score on presentation and a higher neurological grade at 21–30 days for surgically treated dogs (*r* = 0.44, *p* = 0.001). Furthermore, a higher neurological grade on presentation was strongly associated with a longer recovery time for surgically treated subjects (*r* = 0.620, *p* > 0.001). Clinical scores decreased overtime (*p* < 0.001) both for surgically and medically treated patients. Surgically treated patients had a median clinical score of 1 at presentation (range: 1–4), 0 at 3–7 days (range: 0–3), 0 at 21–30 days (range: 0–2), 0 at 6 months (range: 0–2). Medically treated dogs had a median clinical score of 1 at presentation (range: 1–2), 0 at 3–7 days (range 0–3), 0 at 21–30 days (range: 0–1), 0 at 6 months (range: 0–0).

**Table 3:** Comparison of the neurological grade, outcome, and recovery time between the surgical and medical management group.

	*n*	Surgical	Medical	*p*
Grade at presentation	80	1 (1–4)	1 (1–2)	0.308
Grade at 3–7 days	71	0 (0–3)	0 (0–3)	0.406
Grade at 21–30 days	64	0 (0–2)	0 (0–1)	0.941
Grade at 6 months	12	0 (0–2)	0 (0–0)	0.655
Recovery time (days)	71	0 (0–28)	0 (0–1)	0.400

*n*, number of patients for which this information was available; *p*, non-parametric Mann–Whitney Test.

The recovery time was significantly longer in dogs with onset of clinical signs of less than 24 h or between 24 and 48 h compared to dogs with an onset of more than 48 h (3.1 ± 6.5 days versus 1.6 ± 6.2, *p* < 0.001). A longer onset (> 48 h) was poorly associated with a shorter recovery time for dogs treated surgically (*r* = −0.310, *p* = 0.021). For surgically treated patients, there was a strong direct association between a higher clinical score at presentation and a longer recovery time (*r* = 0.620 and *p* < 0.001) and a poor direct association between a higher SCC and a longer recovery time (*r* = 0.340 and *p* = 0.008). It was not possible to perform statistical tests for the recovery time of medically treated dogs due to the low number of cases.

Information on outcome at 30 days (successful versus unsuccessful) was available for 66/80 (83%) of dogs: a successful outcome was obtained in 65/66 dogs (98%) and 53/66 (80%) of dogs were considered “normal.” There was no significant difference between the signalment, clinical presentation and MRI findings of medically versus surgically treated dogs that were deemed normal at 30 days. Among the 13/66 dogs (20%) that failed to return to normal, 4/13 (30%) were treated medically. These dogs all presented with clinical signs >48 h and were categorized as either grade 1 (3/4, 75%) or grade 2 (1/4, 25%) on presentation. Two out of 4 dogs (50%) were diagnosed with a foraminal IVDE without spinal cord compression. The three dogs presenting as grade 1 were reported to have either improved (grade 0, 2/3, 66%) or remained neurologically static (grade 1, 1/3, 33%) at 21–30 days from their diagnosis. The reason for these three dogs not being classified as “normal” at 30 days was the persistence of cervical hyperesthesia when analgesia was discontinued. The dog presenting as grade 2 had improved to grade 0 at 3–7 days from diagnosis. However, the presence of mild pelvic limb ataxia was found at 30 days and therefore this patient was not classified as normal. The remaining 9/13 (70%) dogs were treated surgically (2/9, 22%, dorsal laminectomy; 7/9, 77%, ventral slot). These patients were classified as grade 2 (5/9, 55%), grade 3 (1/9, 11%) or grade 4 (3/9, 44%) on initial presentation with mostly an onset of clinical signs >48 h (7/9, 77%). One of these patients originally presented as a grade 2 and improved to a grade 1 at the 3–7 days check point after surgery. However, the dog then suffered a deterioration of clinical signs at was euthanized at 21–30 days, without repeating investigations. One patient was classified as a grade 2 at the time of original presentation and remained neurologically static at 30 days. The remaining seven patients showed an improvement in their neurological examination of at least one grade at 30 days, however, 3/7 (43%) remained a grade 2. Four out of seven (57%) patients were classified as a grade 0 at 30 days, however, they were still requiring oral analgesia and therefore they could not be classified as normal.

There was no recurrence of clinical signs within 30 days from their original presentation for any of the dogs included in the study. Recurrence of clinical signs after 30 days from their original presentation was only experienced in five dogs: three of the surgically treated dogs (3/52, 6%) and two of the medically treated dogs (2/13, 15%). Details of this group of patients have been summarized in Table 4. None of these patients underwent a repeated MRI scan of the cervical vertebral column. It was not possible to perform statistical tests for this group of patients due to the low number of cases leading to lack of statistical significance.

**Table 4:** Summary of clinical presentation and outcome in the five patients experiencing a recurrence of clinical signs.

	Onset	Grade	Site of IVDE	Foraminal component?	Treatment	Grade at 3–7 days	Grade at 21–30 days	Details on recurrence
1	> 48 h	1	C3-C4	No	Surgical	0	0	Cervical hyperesthesia 8 months after original presentation, returned to normal with medical management for presumed IVDE (no imaging done)
2	> 48 h	1	C2-C3	No	Surgical	0	0	Cervical hyperesthesia and left forelimb lameness 3 years after original presentation, returned to normal with medical management for presumed IVDE (no imaging done)
3	> 48 h	2	C3-C4	No	Surgical	0	0	Cervical hyperesthesia 72 days after surgery; resolved with medical management for presumed IVDE (no imaging done)
4	< 24 h	1	C2-C3	No	Medical	0	0	Mild cervical hyperesthesia 9 months after original presentation; resolved with medical management for presumed IVDE (no imaging done)
5	> 48 h	1	C5-C6	Yes	Medical	0	0	Cervical hyperesthesia 3 months after original presentation; resolved with medical management for presumed IVDE (no imaging done)

Dogs treated medically that underwent physiotherapy (2/18, 11%) compared to those that did not receive physiotherapy had a longer hospitalization, shorter recovery time and a lower neurological score at 21–30 days (*p* < 0.001). Surgical cases treated with physiotherapy (19%; 12/62) had a longer hospitalization time (*p* = 0.012) and higher scores at 3–7 and at 21–30 days (*p* < 0.001). Results are summarized in Table 5.

**Table 5:** Influence of physiotherapy on hospitalization length, outcome and recovery time in medically versus surgically treated patients.

Physiotherapy	Medical Group			Surgical Group		
	Yes (*n* = 2)	No (*n* = 16)	*p*	Yes (*n* = 12)	No (*n* = 50)	*p*
Hospitalization (days)	4 (4–4)	0 (0–4)	**<0.001**	3 (0–5)	1 (0–9)	**0.012**
Outcome 3–7 days (Score 0–5)	1.5 (0–3)	0 (0–2)	0.636	1 (0–3)	0 (0–3)	**<0.001**
Outcome 21–30 days (Score 0–5)	0 (0–0)	0 (0–1)	**<0.001**	0 (0–2)	0 (0–2)	**<0.001**
Recovery Time (days)	0 (0–0)	0 (0–1)	**<0.001**`	0 (0–28)	0 (0–27)	0.165

*p*, non-parametric Mann–Whitney Test. The values of statistical significance in bold.

## 4 Discussion

This retrospective study describes the clinical presentation, magnetic resonance imaging findings and the outcome of eighty Dachshunds diagnosed with cervical IVDE.

In the present study population, smooth-haired Dachshunds, as well as neutered females and entire males were over-represented. A recent online survey (“DachsLife 2018 | Dachshund Health UK”) revealed that miniature smooth-haired Dachshunds were the most common size and coat type in the entire Dachshund population included in the survey. When looking at gender distribution, the same survey highlighted a higher percentage (54%) of entire males compared to entire females (43.8%). We therefore believe that the over-representation of smooth haired Dachshunds and entire males largely reflects their high prevalence among the entire population of Dachshunds rather than an actual predisposition for these two categories. The high prevalence of neutered female dogs in our study seems in line with what previously published by Dorn and Seath (24) where neutered female Dachshunds were found to be at higher risk of intervertebral disc herniation compared to entire females. However, a more recent study focusing on the neutering status of female Dachshunds diagnosed with thoracolumbar intervertebral disc extrusion (25) has actually highlighted how there was no significant difference between age, BCS, clinical presentation and length and degree of spinal cord compression in neutered versus entire female Dachshunds. The median age at diagnosis in our study was seven years. This is in accordance with previous data in dogs with cervical IVDE (4). Another study (26) demonstrated a positive association between obesity and IVDE in several dog breeds. Interestingly, our study did not reveal a significant association between BCS and cervical IVDE in dogs. However, it has to be considered that, due to the retrospective nature of the study, information on BCS was only available for 43/80 dogs and this, together with the fact that most dogs were classified as 5/9 on the BCS, might have contributed to the lack of statistical significance between the two parameters.

In our study, ambulatory dogs (grade 1 and grade 2) were more frequent, in agreement with previous literature (4, 6). In reviewing the data, the authors identified a discrepancy between the neurolocalization and the neurological grade of some of the dogs included in the study. In particular, there were 44/80 dogs classified as a grade 1 but only 34/80 dogs neurolocalized as “cervical hyperesthesia only.” The remaining 10/80 Dachshunds presenting with cervical hyperesthesia (grade 1) had been assigned a neurolocalization to either “C1-C5 spinal cord segments” (8/10) or “C6-T2 spinal cord segments” (2/10) as they did not present with any obvious gait abnormalities, however, they displayed postural reaction deficits or decreased withdrawal reflexes, respectively. There was a relatively low number of dogs presenting with nerve root signatures similarly to other studies (3, 16, 27).

A foraminal IVDE was reported only in 18 cases, in line with current literature suggesting that foraminal IVDE is to be considered a relatively rare condition (3). In our study there was no significantly different prevalence for a particular site, contrary to what was reported in previous studies where C6-C7 appeared the most common site for foraminal IVDE (19, 28). Interestingly, our study revealed a significantly higher prevalence of left-sided foraminal IVDE. To the authors’ knowledge, this predisposition has not been previously reported. Given the lack of an obvious neuro-anatomical explanation for a foraminal IVDE to favor this side, its clinical significance remains uncertain. Further studies focusing on dogs with foraminal IVDE are needed to confirm this finding. In our study, only 25% of the dogs presenting with nerve root signatures were diagnosed with a foraminal IVDE on MRI. Foraminal IVDE can be challenging to diagnose; however, a study on 13 dogs with foraminal IVDE (19) showed how MRI was able to detect a foraminal IVDE on T2W transverse sequences in all patients. Given that in our study, T2W transverse sections were always carried out on the area of interest, it seems unlikely that a foraminal IVDE could have been missed. However, given the retrospective and multicenter nature of our study, it was not possible to include further MRI sequences such as Half fourier Single-shot Turbo spin-Echo (HASTE) or Short-Tau Inversion Recovery (STIR) which might have been useful in evaluating the presence of foraminal IVDE. Nevertheless, this finding could indicate that a dynamic component may play a role in the onset of “nerve root signature” in these patients and does not necessarily correlate to imaging findings suggestive of intervertebral foramen involvement. However, further studies focusing on patients presenting with “nerve root signature” are needed to better evaluate this observation.

The most commonly intervertebral disc space affected in our Dachshund population was C2-C3 (reported in 38% of dogs) as previously reported (5, 6), especially in chondrodystrophic breeds (5). The caudal cervical vertebral column has formerly been identified as a less common location (6) for IVDE and this has also been the authors’ experience in this population of Dachshunds where IVDE in the caudal cervical vertebral column (from C6 to T2) was reported only in 10/80 patients. The reason for this prevalence for the cranial cervical vertebral column in Dachshunds is currently unclear. A possible explanation lies in the unique anatomical conformation of this breed (longer neck compared to other non-chondrodystrophic breeds) resulting in different biomechanics and different load between the cranial and caudal cervical vertebral column. This had been already suggested by Chai et al. (29) when discussing the unusual prevalence for the C6-C7 IVDE in Pekingese dogs, however, further studies focusing on the biomechanics of the cervical vertebral column in chondrodystrophic breeds would be needed to prove this.

When evaluating the degree of IVD degeneration using the Pfirrmann grading system, there was a significantly higher prevalence for grades 3 and 4. This is in accordance with what was previously described by Reunanen et al. (30) in healthy Dachshunds. The median SCC in our study was 28%. This is similar to Ryan et al. (16), where dogs with cervical IVDE had a median SCC of 26%. Unsurprisingly, the signal intensity of the extruded intervertebral disc material in our study was homogenously T2W hypointense compared to grey matter in the majority of cases, as previously described (20). Although predominately T2W hypointense, the signal intensity of the extruded intervertebral disc material did not influence the treatment choice in our study. The presence of T2W intramedullary hyperintensity in dogs with IVDE has been associated with necrosis, myelomalacia, intramedullary hemorrhage, inflammation and edema and has been found to be negatively associated with the patient’s outcome (17). In our population, the presence of T2W intramedullary hyperintensity was found in 59/80 cases. In our study, not only was there no significant difference between the presence of T2W intramedullary hyperintensity and the treatment choice (medical versus surgical) but there was also no significant difference between the outcome of patients with or without T2W intramedullary hyperintensity. However, the length of hospitalization was significantly shorter in dogs without T2W intramedullary hyperintensity compared to dogs with T2W intramedullary hyperintensity on MRI. The authors hypothesized that this finding could be explained by the fact that patients with a T2W intramedullary hyperintensity displayed more severe clinical signs, however, this was not demonstrated in our study. The authors of this study only evaluated the presence/absence of T2W intramedullary hyperintensity and not its extension which might have provided further useful information.

Most of the dogs included in our study underwent surgical treatment (77.5%) rather than medical treatment (22.5%). The therapeutic choice did not appear to be influenced by the onset of clinical signs or by the clinical score on presentation. However, dogs with a compressive IVDE without a foraminal component were more likely to be treated surgically in our study. Treatment choice could be influenced by many factors including the clinician’s personal preference and owners’ financial and personal circumstances and therefore it may be a difficult parameter to evaluate. The authors would have expected that surgical treatment would have been preferred for patients with a highly compressive cervical IVDE (i.e., with a higher SCC), however, this was not the case in our study as treatment choice was also not influenced by the degree of SCC. This might also be influenced by a lack of strong correlation between neurological signs and degree of spinal cord compression, as recently suggested by Bach et al. (31). Patients treated surgically spent on average, a longer time in the hospital compared to medically treated dogs. Furthermore, in the surgically treated group, a higher BCS on presentation and a higher mean SCC calculated on MRI were directly associated with a longer hospitalization time while age at presentation was inversely correlated to the length of hospitalization. The longer hospitalization for these surgically treated patients compared to medically managed dogs may be a result of the post-operative analgesia which usually involves intravenous opioid administration. On the contrary, some of the medically managed patients were treated on an outpatient basis only with oral medications. Intuitively, older dogs and dogs with a higher BCS are likely to require more nursing care and therefore may have a prolonged hospitalization time. As expected, a higher clinical score at presentation was directly associated with a longer time in the hospital and this was found in both medically and surgically treated dogs. The length of hospitalization was also significantly shorter in dogs without a foraminal IVDE. A longer hospitalization duration may be the result of longer analgesia, supportive care and monitoring required for more severely affected dogs or dogs with a higher degree of cervical hyperesthesia (as could be expected in dogs with a foraminal IVDE). The lack of information on treatment received by the dogs included in the study prior to referrals also represents a severe limitation as it could have also influenced the hospitalization length for these patients.

Our study also revealed that the recovery time was significantly longer in dogs with an onset of clinical signs of <24 h or between 24 and 48 h compared to dogs with an onset >48 h. This was also reported in a previous study (32) focusing on surgically treated dogs. Furthermore, in our study, dogs with a longer (> 48 h) onset of clinical signs treated surgically were also reported to have a better outcome at the 3-7-day mark, compared to dogs with onset <24 h or between 24 and 48 h. While there was no significant difference in the outcome at later time points (at 21–30 days and six months), the authors hypothesized that dogs with an onset of clinical signs <24 h or between 24 and 48 h were most likely presenting with a more severe neurological grade, compared to dogs with an onset >48 h leading to a worse outcome at the 3-7-day mark. However, when looking at the clinical score on presentation of dogs with an onset <24 h or between 24 and 48 h versus dogs with an onset >48 h in our study, there was no significant difference between the two groups. Therefore, no obvious explanation for this finding could be extrapolated from our study.

There is limited information in the current veterinary literature regarding the role of physiotherapy in the management of dogs with cervical IVDE. Only one recent study (33) focused on physical rehabilitation for dogs with cervical IVDE treated surgically and highlighted how this can be beneficial for the patients and improve the success rate of the surgery. In our study, the beneficial effect of physiotherapy was mainly seen in medically managed patients as they showed a shorter recovery time and a lower neurological score at 21–30 days. Conversely, in the surgically treated group, dogs that underwent physiotherapy displayed higher scores at the 3–7 and 21-30-day intervals. Both medically and surgically treated Dachshunds that received physiotherapy had a longer hospitalization time. This is likely the result of the fact that physiotherapy is more likely to be advised in dogs with a higher clinical score on presentation which are expected to spend a longer time in the hospital and retain a higher clinical score at the follow-up. A limitation to our study was the lack of standardization of physiotherapy protocols, both in terms of frequency and techniques used, due to the retrospective and multicenter nature of the study. Therefore, even though it is interesting to highlight that physiotherapy had a positive effect in some of the patients of the study, a prospective study with a standardized physiotherapy protocol would actually be needed to draw some more useful conclusions.

One of the most interesting results of our study was that there was no difference between the therapeutic choice (surgical versus medical treatment) and the outcome of Dachshunds with cervical IVDE. The neurological grade of the patients included in our study significantly decreased over time compared to their original presentation, both for the surgically treated and the medically treated group. However, as expected, dogs with a higher clinical score on presentation retained a higher clinical score at the 3–7 days and 21–30 days marks and had an overall longer recovery time. Furthermore, there were no significant difference between medically treated and surgically treated dogs in terms of successful outcome and return to normal. These results are encouraging, as it would appear that for Dachshunds diagnosed with cervical IVDE, medical management could represent a valid treatment option and may allow a successful outcome without the risks and financial burden that a surgical procedure usually entails. Of course, the clinician should expect a longer recovery time for more severely affected dogs. However, it has to be specified that the number of surgically treated patients in our study was higher than the medically treated dogs (62 versus 18 dogs) and also that treatment was decided by the clinician rather than being assigned randomly. Therefore, results could be subject to bias and it is not possible to make a comparison between surgical and medical treatment. No studies comparing surgical versus medical management of dogs diagnosed with cervical IVDE could be found in the current veterinary literature. Surgical treatment for cervical IVDE has been previously reported to be associated with a variable degree of successful outcome, from 64% (34) to 100% (35). There are fewer reports of outcomes in medically treated dogs with cervical IVDE, however, one study (36) suggested successful outcomes in 48.9% of dogs.

In our study, recurrence of clinical signs was experienced in five dogs; three of the surgically treated dogs (6%) and two of the medically treated dogs (15%). As summarized in Table 4, a recurrence was only presumed as these patients did not undergo any advanced diagnostic imaging. This obviously represents a limitation to our study as it would have been interesting to have confirmed a diagnosis of cervical IVDE for these patients and to have looked at the site of IVDE. Our results may suggest that Dachshunds with a compressive IVDE treated surgically were less likely to suffer a recurrence of clinical signs, however, the smaller number of medically treated patients in our study represents an inherent bias. Our recurrence rate appeared less than what was previously reported in veterinary literature. For example, a recent study on the recurrence of signs consistent with cervical IVDE in 119 dogs (37) reported a recurrence rate of 33% for surgically managed dogs and 36% for medically managed dogs between 1 to 112 months following diagnosis. In the study by Argent et al., Dachshunds represented only 8% of the entire population and therefore, although the results of our study may suggest that Dachshunds have a smaller risk of recurrence compared to the general canine population, care needs to be taken in interpreting these results. Further studies involving a larger group of Dachshunds are necessary to better assess the recurrence rate in this breed.

Limitations of this study include its retrospective and multicenter nature. Furthermore, data regarding size and coat type for these patients might be inaccurate as this information was provided by the owners. A larger study population with an even number of medically versus surgically treated dogs would probably be needed to evaluate the outcome of these patients. In our study the definition for “successful outcome” was decided by the authors during study design and prior to collecting the data; given the majority of cases presented as a grade 1 or grade 2, this inevitably represented a limitation and prevented from evaluating further patients that were already ambulatory on presentation. To compensate this, the neurological grade at different time intervals was evaluated as the authors believed it would offer a better understanding of the clinical progression of the patients included in the study.

In conclusion, this study highlighted characteristics of cervical IVDE in Dachshunds that can be of interest to the veterinarians managing these cases. Dachshunds with a cervical IVDE are mostly younger than seven years of age and have a duration of clinical signs >48 h. These patients usually present ambulatory (neurological grade 1 or 2) and without nerve root signature. In addition, this data confirms that the most affected intervertebral disc space is C2-C3 while foraminal IVDEs are rare. Surgery was the most favored treatment option for these patients. The recovery time appears longer in dogs with an onset of clinical signs <24 h or 24 to 48 h compared to those with an onset >48 h. Furthermore, a higher clinical score at presentation and a higher SCC ratio on MRI appear associated with a longer recovery in surgically treated patients. The outcome for Dachshunds with cervical IVDE is generally to be considered successful. No association between the therapeutic choice (medical versus surgical) was observed for the dogs included in this study.

## Data Availability

The original contributions presented in the study are included in the article/Supplementary material, further inquiries can be directed to the corresponding author.

## References

[1] Bergknut N Smolders LA Grinwis GCM Hagman R Lagerstedt A-S Hazewinkel HAW. Intervertebral disc degeneration in the dog. Part 1: anatomy and physiology of the intervertebral disc and characteristics of intervertebral disc degeneration. Vet J. (2013) 195:282–91. doi: 10.1016/j.tvjl.2012.10.024, PMID: 23177522

[2] Klesty A Forterre F Bolln G. Outcome of intervertebral disk disease surgery depending on dog breed, location and experience of the surgeon: 1113 cases. Tierarztl Prax Ausg K Klientiere Heimtiere. (2019) 47:233–41. doi: 10.1055/a-0948-9187, PMID: 31434123

[3] Brisson BA. Intervertebral disc disease in dogs. Vet Clin North Am Small Anim Pract. (2010) 40:829–58. doi: 10.1016/j.cvsm.2010.06.001 20732594

[4] Cherrone KL Dewey CW Coates JR Bergman RL. A retrospective comparison of cervical intervertebral disk disease in Nonchondrodystrophic large dogs versus small dogs. J Am Anim Hosp Assoc. (2004) 40:316–20. doi: 10.5326/0400316, PMID: 15238562

[5] Hakozaki T Iwata M Kanno N Harada Y Yogo T Tagawa M. Cervical intervertebral disk herniation in chondrodystrophoid and nonchondrodystrophoid small-breed dogs: 187 cases (1993–2013). J Am Vet Med Assoc. (2015) 247:1408–11. doi: 10.2460/javma.247.12.1408, PMID: 26642135

[6] Sharp N.J. Wheeler S.J. (2005). Cervical disc disease, in: small animal spinal disorders. Amsterdam: Elsevier, pp. 93–120.

[7] Prata RG Felts JF. Cervical disk disease in the dog—intraforaminal and lateral extrusions. J Am Anim Hosp Assoc. (1983) 19:755–60.

[8] Aikawa T Shibata M Asano M Hara Y Tagawa M Orima H. A comparison of thoracolumbar intervertebral disc extrusion in French bulldogs and dachshunds and association with congenital vertebral anomalies: IVDE in French bulldogs and dachshunds. Vet Surg. (2014) 43:301–7. doi: 10.1111/j.1532-950X.2014.12102.x, PMID: 24433331

[9] Cardy TJA Tzounos CE Volk HA De Decker S. Clinical characterization of thoracolumbar and lumbar intervertebral disk extrusions in English cocker spaniels. J Am Vet Med Assoc. (2016) 248:405–12. doi: 10.2460/javma.248.4.405, PMID: 26829272

[10] DachsLife (2018). Dachshund health UK [WWW document], Dachshund-Health-Uk. Available online at: https://www.dachshundhealth.org.uk/dachslife-2018 (Accessed December 18, 2023)

[11] Palus V Stehlik L Necas A Srnec R Urbanova L Lu D. Cervical intervertebral disc disease in 60 Yorkshire terriers. Front Vet Sci. (2023) 10:1148802. doi: 10.3389/fvets.2023.1148802, PMID: 37252381 PMC10213352

[12] Laflamme D. Development and validation of a body condition score system for dogs. Canine Pract. (1997):10–5.

[13] Dolera M Malfassi L Marcarini S Mazza G Sala M Carrara N. Hydrated nucleus pulposus extrusion in dogs: correlation of magnetic resonance imaging and microsurgical findings. Acta Vet Scand. (2015) 57:58. doi: 10.1186/s13028-015-0151-x, PMID: 26407812 PMC4583177

[14] Tirrito F Cozzi F Bonaldi M Conti S Lombardo R. Agreement of surgeon’s perception of the effectiveness of spinal cord decompression with findings on postoperative magnetic resonance imaging for dogs surgically treated for intervertebral disk extrusion. J Am Vet Med Assoc. (2020) 256:210–9. doi: 10.2460/javma.256.2.210, PMID: 31910078

[15] De Albuquerque Bonelli M Da Costa RC. Clinical and magnetic resonance imaging characterization of cervical spondylomyelopathy in juvenile dogs. J Vet Intern Med. (2019) 33:2160–6. doi: 10.1111/jvim.15602, PMID: 31469206 PMC6766523

[16] Ryan TM Platt SR Llabres-Diaz FJ McConnell JF Adams VJ. Detection of spinal cord compression in dogs with cervical intervertebral disc disease by magnetic resonance imaging. Vet Rec. (2008) 163:11–5. doi: 10.1136/vr.163.1.11, PMID: 18603629

[17] Olby NJ Da Costa RC Levine JM Stein VM The Canine Spinal Cord Injury Consortium (CANSORT SCI). Prognostic factors in canine acute intervertebral disc disease. Front Vet Sci. (2020) 7:596059. doi: 10.3389/fvets.2020.596059 33324703 PMC7725764

[18] Da Costa RC De Decker S Lewis MJ Volk H The Canine Spinal Cord Injury Consortium (CANSORT-SCI). Diagnostic imaging in intervertebral disc disease. Front. Vet. Sci. (2020) 7:588338. doi: 10.3389/fvets.2020.588338, PMID: 33195623 PMC7642913

[19] Bersan E McConnell F Trevail R Behr S De Decker S Volk HA. Cervical intervertebral foraminal disc extrusion in dogs: clinical presentation, MRI characteristics and outcome after medical management. Vet Rec. (2015) 176:597–7. doi: 10.1136/vr.102851, PMID: 25745084

[20] Mai W. Diagnostic MRI in dogs and cats. Boca Raton, FL: CRC Press (2018).

[21] Bergknut N Auriemma E Wijsman S Voorhout G Hagman R Lagerstedt A-S. Evaluation of intervertebral disk degeneration in chondrodystrophic and nonchondrodystrophic dogs by use of Pfirrmann grading of images obtained with low-field magnetic resonance imaging. Am J Vet Res. (2011) 72:893–8. doi: 10.2460/ajvr.72.7.893, PMID: 21728849

[22] Olby NJ Moore SA Brisson B Fenn J Flegel T Kortz G. ACVIM consensus statement on diagnosis and management of acute canine thoracolumbar intervertebral disc extrusion. J Vet Intern Med. (2022) 36:1570–96. doi: 10.1111/jvim.16480, PMID: 35880267 PMC9511077

[23] Lewis MJ Jeffery ND Olby NJ the Canine Spinal Cord Injury Consortium (CANSORT-SCI). Ambulation in dogs with absent pain perception after acute thoracolumbar spinal cord injury. Front Vet Sci. (2020) 7:560. doi: 10.3389/fvets.2020.00560, PMID: 33062648 PMC7479830

[24] Dorn M Seath IJ. Neuter status as a risk factor for canine intervertebral disc herniation (IVDH)in dachshunds: a retrospective cohort study. Canine Genet. Epidemiol. (2018) 5:11. doi: 10.1186/s40575-018-0067-7 30459956 PMC6236875

[25] Doeven L Cardy T Crawford AH. Investigation of neutering status and age of neutering in female dachshunds with thoracolumbar intervertebral disc extrusion. J Small Anim Pract. (2024):jsap.13733. doi: 10.1111/jsap.13733 38622029

[26] Packer RMA Hendricks A Volk HA Shihab NK Burn CC. How long and low can you go? Effect of conformation on the risk of thoracolumbar intervertebral disc extrusion in domestic dogs. PLoS One. (2013) 8:e69650. doi: 10.1371/journal.pone.0069650, PMID: 23894518 PMC3722130

[27] Batcher K Dickinson P Giuffrida M Sturges B Vernau K Knipe M. Phenotypic effects of FGF4 Retrogenes on intervertebral disc disease in dogs. Genes. (2019) 10:435. doi: 10.3390/genes10060435, PMID: 31181696 PMC6627552

[28] Schachar J Bocage A Nelson NC Early PJ Mariani CL Olby NJ. Clinical and imaging findings in dogs with nerve root signature associated with cervical intervertebral disc herniation. J Vet Intern Med. (2024) 38:1111–9. doi: 10.1111/jvim.16982, PMID: 38216520 PMC10937489

[29] Chai O Harrosh T Bdolah-Avram T Mazaki-Tovi M Shamir MH. Characteristics of and risk factors for intervertebral disk extrusions in Pekingese. J Am Vet Med Assoc. (2018) 252:846–51. doi: 10.2460/javma.252.7.846, PMID: 29553897

[30] Reunanen VLJ Jokinen TS Hytönen MK Junnila JJT Lappalainen AK. Evaluation of intervertebral disc degeneration in young adult asymptomatic dachshunds with magnetic resonance imaging and radiography. Acta Vet Scand. (2023) 65:42. doi: 10.1186/s13028-023-00702-0, PMID: 37752484 PMC10523717

[31] Bach FS Mai W Weber LFS Villanova Junior JA Bianchi De Oliveira L Montiani-Ferreira F. Association between spinal cord compression ratio in magnetic resonance imaging, initial neurological status, and recovery after ventral slot in 57 dogs with cervical disc extrusion. Front. Vet. Sci. (2023) 9:1029127. doi: 10.3389/fvets.2022.1029127, PMID: 36686187 PMC9853044

[32] Seim H Prata R. Ventral decompression for the treatment of cervical disk disease in the dog: a review of 54 cases [surgery]. J Am Anim Hosp Assoc. (1982)

[33] Jeong I-S Rahman MM Choi G-C Seo B-S Lee G-J Kim S. A retrospective study of canine cervical disk herniation and the beneficial effects of rehabilitation therapy after ventral slot decompression. Vet Med. (2019) 64:251–9. doi: 10.17221/114/2018-VETMED

[34] Hillman RB Kengeri SS Waters DJ. Reevaluation of predictive factors for complete recovery in dogs with nonambulatory tetraparesis secondary to cervical disk herniation. J Am Anim Hosp Assoc. (2009) 45:155–63. doi: 10.5326/0450155 19570897

[35] Gill PJ Lippincott CL Anderson SM. Dorsal laminectomy in the treatment of cervical intervertebral disk disease in small dogs: a retrospective study of 30 cases. J Am Anim Hosp Assoc. (1996) 32:77–80. doi: 10.5326/15473317-32-1-77, PMID: 8963741

[36] Levine JM Levine GJ Johnson SI Kerwin SC Hettlich BF Fosgate GT. Evaluation of the success of medical Management for Presumptive Cervical Intervertebral Disk Herniation in dogs. Vet Surg. (2007) 36:492–9. doi: 10.1111/j.1532-950X.2007.00296.x, PMID: 17614931

[37] Argent V Perillo R Jeffery N Freeman P. Recurrence of signs consistent with cervical intervertebral disc extrusion in dogs. J Small Anim Pract. (2022) 63:454–9. doi: 10.1111/jsap.13480, PMID: 35146745 PMC9303190

