# Supplementary material for: Clinical presentation, magnetic resonance imaging findings, and outcome of 80 Dachshunds with cervical intervertebral disc extrusion
Source: Front Vet Sci. 2024 Aug 29;11:1438300. doi: 10.3389/fvets.2024.1438300 (PMC11391899; doi:10.3389/fvets.2024.1438300)
Supplement: Supplementary file 1 [file Table_1.pdf]

frontiers

Clinical presentation, magnetic resonance imaging findings and outcome of 80 Dachshunds with cervical intervertebral disc extrusion

Supplementary Table 1. Influence of age at diagnosis, weight, body condition score (BCS), onset of clinical signs, clinical score at presentation, degree of spinal cord compression (SCC) or degree of IVD degeneration (Pfirrmann grade) of the extruded IVD on hospitalization length, outcome or recovery time

	**Hospitalization (days)**		**Outcome 3-7 days**		**Outcome 21-30 days**		**Outcome at 6 months**		**Recovery Time**	
	**Surgical**	**Medical**	**Surgical**	**Medical**	**Surgical**	**Medical**	**Surgical**	**Medical**	**Surgical**	**Medical**
**Age at time of diagnosis (years)**	-0.36**	0.20	-0.14	-0.21	-0.27	-0.35	-0.30	.	0.06	0.26
**Weight (kg)**	0.09	-0.29	0.18	-0.07	0.03	0.48	0.14	.	0.16	0.13
**BCS**	0.49**	0.55	0.15	0.52	-0.45*	.	.	.	-0.03	0.68
**Onset (days)**	0.15	-0.14	-0.43***	-0.04	-0.27	0.49	-0.18	.	-0.31*	-0.09
**Clinical score at presentation**	0.36**	0.47*	0.67***	0.63*	0.44**	-0.25	0.33	.	0.62***	-0.21
**SCC**	0.31*	0.13	0.37**	-0.35	0.23	.	0.17	.	0.34**	0.71
**Pfirrmann grade of extruded IVDE**	0.23	-0.16	-0.05	0.31	-0.10	0.51	-0.07	.	-0.02	0.00

Spearman rank correlation; *0.01<P<0.05 ; **0.001<P<0.01; ***P<0.001 ; . not estimable (n<=5)

Supplementary Table 2. Influence of pre-treatment variables (size, sex, neutered status, history of previous spinal problems), neurolocalization, MRI findings (presence/absence of T2W intramedullary hyperintensity, signal intensity on T2W of extruded intervertebral disc material) on hospitalization length, outcome or recovery time

	**Hospitalization (days)**			**Outcome 3-7 days**			**outcome 21-30 days**			**Outcome 6 months**			**Recovery time (days)**		
	**Surgical**	**Medical**	**P**	**Surgical**	**Medical**	**P**	**Surgical**	**Medical**	**P**	**Surgical**	**Medical**	**P**	**Surgical**	**Medical**	**P**
**Size**															
Miniature	3(0-9)	1(0-4)	0.384	0(0-3)	0(0-3)	0.472	0(0-2)	0(0-0)	0.417	0(0-2)	0(0-0)	0.527	0(0-27)	0(0-1)	0.670
Standard	1(0-5)	0(0-0)	**0.001**	0(0-3)	0(0-1)	0.727	0(0-2)	0(0-1)	0.420	0(0-0)	.	.	0(0-28)	0(0-0)	0.341
**Sex**															
FE	1(0-3)	1(0-4)	0.680	0(0-2)	2.5(2-3)	0.052	0(0-2)	0(0-0)	0.479	0(0-2)	0(0-0)	0.564	0(0-0)	0(0-0)	0.999
FN	2(0-5)	0(0-4)	**0.017**	0(0-3)	1(0-2)	0.097	0(0-2)	0(0-1)	0.312	0(0-0)	.	.	0(0-28)	0(0-0)	0.539
ME	1(0-5)	0(0-4)	0.124	0(0-3)	0(0-0)	0.160	0(0-2)	0(0-0)	0.628	0(0-0)	.	.	0(0-20)	0(0-0)	0.211
MN	2(0-9)	0.5(0-1)	0.116	0(0-3)	0(0-0)	0.534	0(0-2)	0(0-0)	0.763	0(0-0)	0(0-0)	0.999	0(0-27)	1(1-1)	0.213
**Neutered**															
No	1(0-5)	0(0-4)	0.232	0(0-3)	0(0-3)	0.929	0(0-2)	0(0-0)	0.441	0(0-2)	0(0-0)	0.655	0(0-20)	0(0-0)	0.197
Yes	2(0-9)	0(0-4)	**0.004**	0(0-3)	1(0-2)	0.244	0(0-2)	0(0-1)	0.459	0(0-0)	0(0-0)	0.999	0(0-28)	0(0-1)	0.843
**Neurolocalization**															
neck pain	1(0-5)	0(0-1)	**0.018**	0(0-1)	0(0-1)	0.167	0(0-0)	0(0-1)	0.061	0(0-0)	.	.	0(0-0)	0(0-0)	0.999
C1-C5	2(0-5)	2(0-4)	0.884	0(0-3)	1.5(0-3)	0.462	0(0-2)	0(0-0)	0.647	0(0-0)	0(0-0)	0.999	0(0-28)	0(0-0)	0.437

	Hospitalization (days)			Outcome 3-7 days			outcome 21-30 days			Outcome 6 months			Recovery time (days)		
	**Surgical**	**Medical**	**P**	**Surgical**	**Medical**	**P**	**Surgical**	**Medical**	**P**	**Surgical**	**Medical**	**P**	**Surgical**	**Medical**	**P**
C6-T2	2(0-9)	1(0-4)	0.068	0(0-3)	2(0-2)	0.650	0(0-2)	0(0-0)	0.376	0(0-0)	0(0-0)	0.617	0(0-21)	0(0-1)	0.778
**Nerve root signature**															
No	2(0-9)	0(0-4)	**0.014**	0(0-3)	1(0-3)	0.162	0(0-2)	0(0-1)	0.908	0(0-2)	0(0-0)	0.705	0(0-28)	0(0-0)	0.178
Yes	1(0-3)	0.5(0-1)	0.099	0(0-2)	0(0-0)	0.363	0(0-0)	0(0-0)	0.999	0(0-0)	0(0-0)	0.999	0(0-2)	0(0-1)	0.549
**Compressive versus foraminal IVDE**															
Compressive	2(0-9)	0(0-1)	**0.002**	0(0-3)	0(0-0)	0.404	0(0-2)	0(0-0)	0.712	0(0-2)	0(0-0)	0.739	0(0-28)	0.5(0-1)	0.290
Foraminal	0(0-4)			1(0-3)			0(0-1)			0(0-0)			0(0-0)		
Mixed	2(0-5)	4(0-4)	0.907	0(0-3)	0(0-2)	0.999	0(0-2)	0(0-0)	0.479	0(0-0)	.	.	0(0-20)	0(0-0)	0.329
**Previous spinal problems**															
No	2(0-9)	0(0-4)	**0.010**	0(0-3)	0(0-2)	0.780	0(0-2)	0(0-1)	0.843	0(0-0)	0(0-0)	0.999	0(0-28)	0(0-1)	0.467
Yes	2(0-4)	0(0-4)	0.165	0(0-2)	2(0-3)	**0.036**	0(0-2)	0(0-0)	0.655	0(0-2)	0(0-0)	0.705	0(0-23)	0(0-0)	0.565
**T2W intramedullary hyperintensity**															
No	1(0-5)	0(0-4)	**0.007**	0(0-3)	0(0-3)	0.457	0(0-2)	0(0-0)	0.481	0(0-2)	0(0-0)	0.705	0(0-27)	0(0-0)	0.227
Yes	3(0-9)	1(0-4)	0.377	0(0-3)	1(0-2)	0.695	0(0-2)	0(0-1)	0.523	0(0-0)	0(0-0)	0.999	0(0-28)	0(0-1)	0.840
**Signal intensity of extruded disc material on MRI (T2W sequences)**															
Heterogeneous	1(0-5)	1(0-4)	0.721	0(0-3)	0(0-2)	0.999	0(0-2)	0(0-1)	0.695	.	.	.	0(0-28)	0(0-0)	0.301
Homogenous	2(0-9)	0(0-4)	**0.002**	0(0-3)	0(0-3)	0.366	0(0-2)	0(0-0)	0.451	0(0-2)	0(0-0)	0.655	0(0-27)	0(0-1)	0.801

FE: female entire, FN: female neutered, ME: male entire, MN: male neutered; IVDE: intervertebral disc extrusion,

Data reported as median (min-max); P: non-parametric Mann-Whitney Test

Supplementary Table 3. MRI findings, treatment and outcome of the sub-group of Dachshunds presenting with cervical hyperesthesia only (grade 1).

**Site of IVDE**	**C2-C3**	**C3-C4**	**C4-C5**	**C5-C6**	**C6-C7**	**C7-T1**
N	21	5	4	8	4	2

**Foraminal component**	**Yes**	**NO**
N	12	32
**T2W signal intensity of extruded disc**	**Heterogeneous**	**Homogeneous**
N	10	34
**Presence of T2W intramedullary hyperintensity**	**Yes**	**No**
N	10	34
**Treatment**	**Medical**	**Surgical**
N	10	34

	**Grade 0**	**Grade 1**	**Lost to follow-up**
**Outcome at 3-7 days**	N= 36	N= 4	N= 4
**Outcome at 21-30 days**	N= 35	N= 1	N= 8
**Return to normal at 30 days?**	**Yes**	**No**	**Lost to follow-up**
N	34	3	7

N = number of dogs
